# Non-coding RNAs in diabetes mellitus and diabetic cardiovascular disease

**DOI:** 10.3389/fendo.2022.961802

**Published:** 2022-09-06

**Authors:** Chengshun Li, Dongxu Wang, Ziping Jiang, Yongjian Gao, Liqun Sun, Rong Li, Minqi Chen, Chao Lin, Dianfeng Liu

**Affiliations:** ^1^ Laboratory Animal Center, College of Animal Science, Jilin University, Changchun, China; ^2^ Department of Hand and Foot Surgery, The First Hospital of Jilin University, Changchun, China; ^3^ Department of Gastrointestinal Colorectal and Anal Surgery, China-Japan Union Hospital of Jilin University, Changchun, China; ^4^ Department of Pediatrics, First Hospital of Jilin University, Changchun, China; ^5^ School of Grain Science and Technology, Jilin Business and Technology College, Changchun, China

**Keywords:** diabetes mellitus, microRNA, long non-coding RNA, circular RNA, cardiovascular disease

## Abstract

More than 10% of the world’s population already suffers from varying degrees of diabetes mellitus (DM), but there is still no cure for the disease. Cardiovascular disease (CVD) is one of the most common and dangerous of the many health complications that can be brought on by DM, and has become the leading cause of death in people with diabetes. While research on DM and associated CVD is advancing, the specific mechanisms of their development are still unclear. Given the threat of DM and CVD to humans, the search for new predictive markers and therapeutic ideas is imminent. Non-coding RNAs (ncRNAs) have been a popular subject of research in recent years. Although they do not encode proteins, they play an important role in living organisms, and they can cause disease when their expression is abnormal. Numerous studies have observed aberrant ncRNAs in patients with DM complications, suggesting that they may play an important role in the development of DM and CVD and could potentially act as biomarkers for diagnosis. There is additional evidence that treatment with existing drugs for DM, such as metformin, alters ncRNA expression levels, suggesting that regulation of ncRNA expression may be a key mechanism in future DM treatment. In this review, we assess the role of ncRNAs in the development of DM and CVD, as well as the evidence for ncRNAs as potential therapeutic targets, and make use of bioinformatics to analyze differential ncRNAs with potential functions in DM.

## Introduction

DM is a metabolic disease characterized by high blood sugar and is mainly classified as type 1 diabetes mellitus (T1DM), type 2 diabetes mellitus (T2DM), gestational diabetes mellitus (GDM), and other less common types (1). T1DM is mainly caused by the destruction of beta cells due to autoimmune abnormalities (2). The main features of T2DM are insulin resistance (IR) and islet β-cell dysfunction (3). Despite advances in medical care, there is still no complete cure for DM. Numerous risk factors for DM have been identified, but the specific mechanisms underlying the development of DM are not yet fully understood (4, 5). Existing studies suggest that numerous signaling pathways involved in the development of DM. Inhibition of antioxidant stress pathways such as Keap1/Nrf2 promotes the progression of DM (6). Endoplasmic reticulum stress pathways, including PERK-ATF4-CHOP, lead to β-cell destruction in DM (7). The abnormal phosphorylation of insulin receptor (INSR) brings about IR (3). In addition, patients with DM are often afflicted with other concomitant diseases, including microvascular diseases such as kidney disease and eye disease, and macrovascular diseases such as CVD and cerebrovascular disease. Of these diseases, CVD has become the leading cause of death in patients with diabetes (8, 9). Various types of DM are considered to be the main cause of CVD, which includes coronary heart disease, peripheral artery disease, and heart failure (10). In recent years, the focus of research on the mechanisms of diabetes and CVD development has shifted to the molecular level, including the study of disturbed epigenetic modifications and abnormal ncRNA expression (11, 12).

Over 98% of human genome expression products are ncRNAs, which mainly include microRNAs (miRNAs) with a length of 19 to 25 bases, long non-coding RNAs (lncRNAs) with a length of more than 200 bases, and ring-loaded RNAs (circRNAs) characterized by a closed-loop structure (13). Although these ncRNAs do not express proteins, they still play an important role in organism. miRNAs can bind to messenger RNAs (mRNAs) whose 3’-untranslated region (3’UTR) complements them, thereby regulating the expression of target genes. In this way, miRNAs are indirectly involved in the regulatory functions of numerous physiological activities. LncRNAs possess non-random short open reading frames (sORFs), and are involved in key processes such as chromatin modification, chromosome recycling, and DNA transcription. Some studies have shown that circRNAs regulated the expression of downstream target genes by binding to miRNAs. However, a recent study has shown that circRNAs can bind to their host genes and directly regulate the expression of the host genes (14). In recent years, it has been found that ncRNAs play an important role in maintaining the normal activities of the body and that abnormal expression of ncRNAs is closely related to the development of many diseases, including DM (15–17). An increasing number of studies have focused on ncRNAs in DM and its complications, suggesting that ncRNAs can interact with insulin (18). Evidence also suggests that ncRNAs may serve as modulators and diagnostic markers of diabetic cardiovascular disease (19–22).

In this review, we summarized some of the evidence regarding the role of ncRNAs in the development of DM and diabetic CVD and their use as therapeutic targets. Related sequencing results revealed differential ncRNAs present in the development of DM and CVD (https://www.ncbi.nlm.nih.gov/geo/). The common DM treatment drug metformin changed the expression level of some ncRNAs after treatment. We also used the data from the Gene Expression Omnibus (GEO) to perform a clustering analysis of target genes of differentially expressed miRNAs in various types of DM. This evidence suggests that ncRNAs play an important role in DM and CVD, and may be the key to treat these diseases in the future.

## Diabetes mellitus

### Type 1 diabetes mellitus

T1DM, also known as “insulin-dependent diabetes mellitus”, is caused by autoimmune destruction of insulin-producing β-cells in the patient’s pancreas and manifests as an absolute deficiency of insulin (23). Mutations in genes such as HLA, INS, CTLA4, and PTPN22 have been identified in this genetically sensitive group of immune system abnormalities, which combined with environmental factors, eventually lead to the development of T1MD (24, 25). During the early development of T1DM, islet autoantigens such as insulin, tyrosine phosphatase IA2, glutamic acid decarboxylase (GAD), and zinc transporter protein 8 (ZNT8) become targets of the immune system (26). Multiple mechanisms exist for the development of T1DM, and ncRNAs play an important role in these mechanisms. Numerous studies have shown that ncRNAs play an important role in immune abnormalities in T1DM, such as elevated miR-34a and decreased miR-146a associated with GAD antibodies (27, 28). miR-143-3p, which is up-regulated in peripheral blood mononuclear cells of T1DM patients, can engage with the inflammatory response by further down-regulating IL-2, TNF-α, and IFN-γ expression through FOSL2 (29). Apoptosis of β-cells is the main manifestation of their disruption. miRNA-203a was found to be elevated in β-cells in a mouse non-obese diabetes (NOD) model (A T1DM animal model), and was verified to regulate β-cell proliferation and apoptosis through IRS2 (30). Abnormal β-cell insulin secretion is also one of the main manifestations of impaired β-cell function in T1DM patients. In a mouse NOD model, LncRNA MALAT1 inhibited insulin secretion by decreasing PDX-1 promoter histone acetylation (31). When β-cells are destroyed, glucose homeostasis is disturbed in patients, leading to acute diseases like ketoacidosis or hyperosmolar coma and secondary complications such as cardiovascular disease and blindness (32). Over the past few decades, it has been observed that the majority of patients with T1DM develop the disease at a young age or in adolescence, causing T1DM to initially be considered as “juvenile diabetes”. s (33). Owing to the discovery of insulin, T1DM has changed from an acute fatal disease to a chronic disease requiring regular exogenous insulin supplementation to keep patients alive (34). However, insulin supplementation is a double-edged sword for T1DM patients, as inappropriate insulin dosage can lead to severe hypoglycemia (35). Several insulin analogs have been developed to avoid such side effects, with improved performance in areas such as speed of function, risk to patient, and convenience (36, 37). Furthermore, a proportion of T1DM patients are misdiagnosed as T2DM in clinical settings and there are extensive limitations to the effectiveness of single insulin therapy (38). Therefore, the search for novel biomarkers and therapeutic ideas for T1DM is of great importance to the quality of life for T1DM patients.

### Type 2 diabetes mellitus

T2DM, also known as “non-insulin-dependent diabetes”, is a common chronic disease that corresponds to T1DM and accounts for more than 90% of the DM patients (https://www.idf.org/). The main features of T2DM are hyperinsulinemia, IR, and defective insulin secretion caused by β-cell failure (39). Consistent with T1DM, T2DM is also caused by a combination of genetic and environmental factors. Genetic polymorphisms in several genes have been observed to be associated with T2DM, and over 200 susceptibility genes have been identified in T2DM, including KLF14, KCNQ1, DUSP9, and FTO (40). Unlike T1DM, unhealthy lifestyle habits such as a high-calorie diet and lack of exercise account for a large proportion of the factors in the development of T2DM (41). As these poor lifestyle habits persist, hyperglycemia and hyperlipidemia, which favor IR and inflammation, emerge and expose β-cells to toxic stresses such as inflammation, endoplasmic reticulum stress, and metabolic/oxidative stress, leading to loss of islet integrity in severe cases (42). There is growing evidence that ncRNAs are extensively involved in these processes. miR-29 promotes the recruitment and activation of circulating monocytes and macrophages in a TRAF3-dependent manner, thereby promoting inflammation (43). Additionally, miRNA-21 induces endoplasmic reticulum stress through activation of mTORC1, leading to β-cell apoptosis (44). Lower lncRNA Eif4g2 has been observed in dysfunctional mouse islets and β-cells, which contributed to apoptosis in β-cells with diminished ability to inhibit oxidative stress (45). T2DM is a lipotoxicity-related disease in which obesity-induced miR-802 impairs insulin transcription by inhibiting NeuroD1 and reduces insulin secretion by inhibiting calcium influx field (46). Another recent study showed that the adipocyte-derived exosome miR-27a induces IR in skeletal muscle by inhibiting PPARγ, which is one of the reasons that obese people are more likely to develop T2MD (47). Although early studies showed that T2DM occurs in older age groups, an increase in unhealthy lifestyle habits associated with modernization has led to the rejuvenation of patients with T2DM. β-cell loss also occurs more rapidly in young patients (10-17 years), resulting in increased early treatment failure in younger patients (48). The current prevention or control of T2DM is usually based on establishing good lifestyle habits, such as a healthy diet and physical exercise (49, 50). Because T2DM is a lipotoxicity-related disease that is associated with an unhealthy lifestyle, it has been widely reported that both exercise and diet can improve T2DM to some extent (51). NcRNAs were observed to be involved in the improvement of T2DM by exercise. For example, miR-143, which causes IR, was observed to be downregulated after aerobic exercise (52). Insulin can also be used to treat T2DM, and it was found that lncRNA LncASIR, a downstream regulator of the insulin signaling pathway, enhances insulin pathway gene transcription in adipocytes during treatment with insulin (53). Therapeutic drugs for T2DM are usually used to balance the blood glucose range through various mechanisms such as improvement of insulin synthesis and regulation of glucose utilization. These drugs mainly include the following categories: metformin and insulin secretion stimulators, alpha-glucosidase inhibitors, thiazolidinediones (TZDs), glucagon-like peptide-1 (GLP-1) analogs, dipeptidyl peptidase 4 (DPP-4) inhibitors, and sodium-dependent glucose co-transport protein inhibitors (SGLT2-Is), all of which have potentially toxic side effects (54, 55). Despite the extensive research on T2DM and the development of novel drugs, T2DM remains a disease that cannot be treated and continues to severely affect humans (56, 57).

### Gestational diabetes mellitus

**Figure 1:**
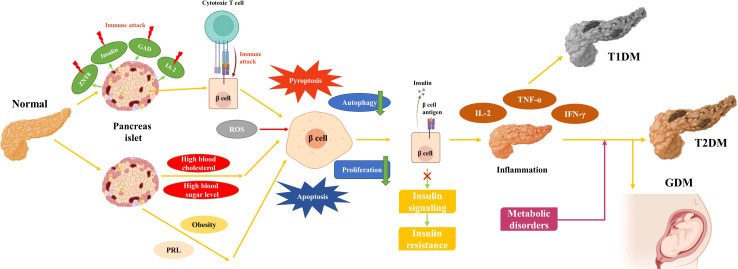
Development of T1DM, T2DM, and GDM.

GDM was initially described as a hyperglycemia caused by poor glucose tolerance first detected during pregnancy (58). It is most recently defined as DM that is not evident before pregnancy but is diagnosed in the middle or late stages of pregnancy (59). GDM is one of the most common diseases during pregnancy, and the International Diabetes Federation has shown that one in six newborns worldwide is affected by GDM (https://www.idf.org/). GDM is very comparable to T2DM, with risk factors including genetic and environmental factors and obesity (60). And like T1DM and T2DM, the development of GDM is also a gradual process (**Figure 1**). Furthermore, women with a history of GDM have a much higher risk of developing subsequent T2DM than women with no history of GDM (61). More than half of patients with a history of GDM develop T2DM within 5-10 years after delivery. GDM can also be harmful to the patient’s newborn and is associated with higher infant mortality, large babies, and premature births (62–64). It has been shown that insulin sensitivity is reduced by more than half in late pregnancy compared to pre-pregnancy. During healthy pregnancy, glucose regulation changes to supply the needs of the developing fetus, and when the pancreatic β-cells are unable to respond properly to this change, it can lead to hyperglycemia (65). Such changes are often due to increases in hormones such as prolactin (PRL) and placental prolactin, the levels of which typically rise during pregnancy. One study has shown that these hormones signal through the prolactin receptor (PRLR) on target cells and that loss of PRLR signaling in pregnant mice leads to loss of MafB expression in β-cells, resulting in loss of proliferation and poor glucose tolerance in β-cells, while non-pregnant mice are not affected (66). GDM also bears similarities to T1DM and T2DM in how it is treated. Firstly, after considering the health of the patient and the fetus, the treatment of GDM prioritizes lifestyle interventions. Glucose-lowering therapy for GDM, which becomes necessary if lifestyle interventions fail, mainly consists of insulin therapy and oral hypoglycemic agents. For patients who do not wish to receive insulin therapy, metformin and glibenclamide are the only oral hypoglycemic agents available for treatment. Due to the long-term effects of GDM on both the pregnant woman and the fetus, and the fact that the potential side effects of most drugs are not known, extreme caution is needed when using these drugs during pregnancy (67).

### Other types of diabetes

In addition to the three most common forms of diabetes mentioned above, there are also other less common forms of diabetes, such as monogenic diabetes and Cystic fibrosis-related diabetes (CFRD).

Monogenic diabetes are caused by a single gene mutation and account for less than 5% of DM patients. Types of monogenic diabetes mainly include Neonatal Diabetes Mellitus (NDM), Maturity-Onset Diabetes of the Young (MODY), and some syndromic forms of diabetes (68). NDM is defined as diabetes diagnosed within six months of birth and usually presents clinically alongside hyperglycemia, growth retardation, and sometimes, dehydration (69). NDM is categorized into temporary (TNDM) and permanent (PNDM), with the distribution roughly equal for each type (69). KCNJ11 and ABCC8, as genes that encode ATP-sensitive potassium channel subunits in pancreatic cells, are the most frequently observed gene defects in PNDM (59). Mutations in the insulin gene (INS) are the second most common cause of PNDM (70). Approximately 70% of TNDMs are caused by abnormalities in chromosome 6q24, and defects in genes such as KCNJ11, ABCC8, and INS have also been found in the remaining cases (71). The current treatment of NDM mainly includes insulin therapy and sulfonylurea therapy (70). MODY is a group of hereditary DM that develops at a young age, shares some of the atypical features of T1DM and T2DM, and lacks β-cell autoimmunity or IR (72). More than 15 MODY-related genes have been identified, and defects in these genes can lead to β-cell dysfunction (73). The most common forms of mutation are GCK, HNF1A, and HNF4A; these genes are prioritized for clinical diagnosis (74). The diagnosis of MODY is very important for treatment, as it determines the treatment strategy. For example, patients with GCK-MODY who do not have other types of DM do not require glucose-lowering therapy except for special circumstances during pregnancy, and do not have macrovascular complications (73). While the most common HNF1A-MODY CVD risk is similar to that of T2DM, which is usually treated with sulfonylureas, the GLP-1 RA used to treat T2DM has recently been shown to be effective in treating HNF1A-MODY as well (75, 76).

CFRD is a specific yet very common form of diabetes in patients with cystic fibrosis (CF) (77). CFRD has few macrovascular complications and causes little to no fatal CVD (78). However, CFRD has remained a major complication of CF in recent years and has significantly increased pulmonary morbidity and mortality in CF patients. The etiology of CFRD is complex, and current evidence suggests that chloride channel defects, oxidative stress, inflammation, and the intestinal proinsulin axis are all involved in the development of CFRD (79). Due to these factors, patients with CF have impaired pancreatic exocrine secretion, followed by fibrosis and fatty infiltration, ultimately leading to structural destruction of the islets (80). It appears that CFRD undergoes islet cell-targeted destruction, however, in contrast to T1DM, this effect is not autoimmune. In contrast, diet interventions for CFRD patients are opposite to those of T2DM patients, as the increased energy consumption requires patients to consume large amounts of calories to maintain their weight and nutrition (81). Existing non-insulin hypoglycemic drugs are not suitable for CFRD. While modulators and enhancers that target cystic fibrosis transmembrane conductance regulators (CFTR) function are being developed, insulin is currently the only recommended drug for the treatment of CFRD (82).

## NcRNAs in diabetes

### MiRNAs

miRNAs are thought to be key factors in post-transcriptional regulation and are first transcribed in the nucleus as pri-miRNAs, which undergo a series of processing before maturing (83). miRNA then binds to Argonaute proteins to form the RNA silencing complex (RISC). miRNAs inhibit translation or induce degradation of target mRNAs by directing RISC to mRNAs with sequence-complementary paired miRNA response elements (MREs) (84). This match between miRNA and target mRNA is usually not strict, which allows for a broader range of miRNA regulation (85). In recent years, researchers have realized that miRNAs play an important role in the development of diseases, including diabetes (86, 87). Inter-ethnic miRNA variants have been reported to be associated with regionally distributed diabetes; for example, miR-196a and miR-423 variants have been observed in T2DM patients in Saudi Arabia (88). Some miRNAs have been observed to be elevated during the development of DM, such as miR-25 and miR-92b (89). In general, these miRNAs with elevated expression promote the development of DM and play a role in the body’s IR, damage to β-cells, and other processes central to the development of diabetes. As research progresses, the specific role of miRNAs in the development of DM is gradually being uncovered. Increasing evidence suggests that miRNAs promote pancreatic β-cell injury in diabetes. MiR-195 promotes pancreatic β-cell dedifferentiation by targeting Mfn2 and impairing Pi3k/Akt signaling in T2MD (90), miRNA-200c targets transcription factor ETV5 to reduce insulin secretion (91), and miR-4431 targets TRIP10/PRKD1 and impairs glucose metabolism (92). There are also some miRNAs that are lost in expression during the progression of DM. The deletion of miR-16 in muscle, for example, leads to impaired insulin sensitivity in men and increased glucose intolerance in women (93).

With advances in sequencing technology, alterations in miRNAs have been identified as a key factor in the study of the mechanism of action of some antidiabetic drugs. As a result, some studies have focused on reversing the abnormal expression of miRNAs with the aim of producing a therapeutic effect on DM. It was shown that increasing the expression of miRNA-494, miRNA-92a, miR-136-5p, and miR-149-5p improved pancreatic β-cell proliferation and insulin secretion (94–96). Furthermore, miR-150-3p and miR-17-5p can protect β-cell function: The former can inhibit β-cell focal death and the latter can alleviate β-cell dysfunction by activating PDX1 signaling (97, 98). miRNA-16-5p was found to be expressed at lower level in T1DM patients and could inhibit high-glucose-induced pancreatic β-cell apoptosis by targeting CXCL10 (99). The downregulation of miR183-3p could treat GDM by reducing skeletal muscle IR (100). Hyperinsulinemia is a typical symptom of T2DM, and studies have shown that exosome-derived miR-26a increases insulin sensitivity by enhancing insulin signaling, thereby reducing hyperinsulinemia (101). miR-17-5p-Mfn1/2-NF-ΚB pathway can exert anti-inflammatory and anti-apoptotic effects in GDM (102). miR-1249-3p has reduced IR and inflammation in a mouse model of T2DM (103). And miRNA-26a can promote regulatory T cells to suppress T1DM (104). Moreover, miR-212/132-enriched extracellular vesicles can be used to promote the differentiation of induced pluripotent stem cells into pancreatic β-cells, and cell replacement therapy for T1DM can be performed using IPSC (105). In conclusion, there is growing evidence that miRNAs have potential anti-diabetic potential in addition to being biomarkers of DM, which may be a major direction for the future treatment of DM (**Table 1**).

**Table 1:** The role of miRNAs in diabetes mellitus.

miRNA	Type of DM	Expression	Effect	Reference
miR-34a	T1DM	Up	Regulate antibody production	(27)
miR-146a	T1DM	Down	Regulate antibody production	(28)
miR-143-3p	T1DM	Up	Promote inflammatory response	(29)
miRNA-203a	T1DM	Up	Adjust the proliferation and apoptosis of β-cells	(30)
miR-29	T2DM	Up	Promote inflammatory response	(43)
miR-21	T2DM	Up	Induce endoplasmic reticulum stress	(44)
miR-802	T2DM	Up	Reduce insulin secretion	(46)
miR-27a	T2DM	Up	Promote insulin resistance	(47)
miR-143	T2DM	Up	Promote insulin resistance	(52)
miR-196A	T2DM	Mutating	–	(88)
miR-423	T2DM	Mutating	–	(88)
miR-25	DM	Up	Induce the apoptosis of β-cells	(89)
miR-92b	DM	Up	Induce the apoptosis of β-cells	(89)
miR-195	T2DM	Up	Promote the dedifferentiation of β-cells	(90)
miRNA-200c	T2DM	Up	Reduce insulin secretion	(91)
mIR-4431	T2DM	Up	Impair glucose metabolism	(92)
miR-16	T2DM	Down	Affect insulin sensitivity and insulin resistance	(93)
miR-494	GDM	Down	Regulate pancreatic cells proliferation and insulin secretion	(94)
miR-92a	T2DM	Down	Regulate pancreatic cells proliferation and insulin secretion	(83)
miR-136-5p	T2DM	Down	Regulate pancreatic cells proliferation and insulin secretion	(96)
miR-149-5p	T2DM	Down	Regulate pancreatic cells proliferation and insulin secretion	(96)
miR-150-3p	T2DM	Down	Reduce β-cells dysfunction	(97)
miR-17-5p	DM	Down	Inhibit the pyrophosis of β-cells	(98)
miR-16-5p	T1DM	Down	Inhibit the apoptosis of β-cells	(99)
miR-182-3p	GDM	Up	Promote insulin resistance	(100)
miR-26a	T2DM	Down	Regulate insulin sensitivity	(101)
miR-17-5p	GDM	Down	Anti-inflammation and anti-apoptosis	(102)
miR-1249-3p	T2DM	Down	Reduce insulin resistance and inflammation	(103)
miR-26a	T1DM	Down	Promote T cell genesis	(104)
miR-212/132	T1DM	–	Induce pluripotent stem cells to differentiate into β-cells	(105)

### LncRNAs

lncRNAs play a role in gene activation and silencing, variable splicing, and post-translational modifications in organisms (106, 107). The competing endogenous RNA (ceRNA) hypothesis proposed in 2011 has greatly enriched the role of lncRNAs in post-transcriptional regulation and has led to increased attention to the physiological role of lncRNAs. The ceRNA hypothesis suggests that lncRNAs can regulate miRNA and target mRNAs by competitively binding to miRNAs (108). lncRNA H19 was found to be associated with diabetes more than a decade ago, but its identity in the study was that of an imprinted gene rather than of a lncRNA (109, 110). The proposal of ceRNA has led to a deeper understanding of the role of lncRNA in various physiological and pathological phenomena, and the role of lncRNA in diabetes has attracted attention in recent years. As mentioned above, miRNAs play an important role in DM, suggesting that lncRNAs may also be involved in the development of DM.

Similar to miRNAs, lncRNAs have been observed to be aberrantly expressed in DM. For example, lncRNA OIP5-AS1 is reduced in GDM, while HOTAIR is highly expressed, both can be used as biomarkers of GDM (111, 112). LncRNA RPL13p5 is highly expressed in GDM patients, forming a co-expression chain with TSC2 gene through PI3K-Akt signaling pathway to promote IR (113). A recent study showed that lncRNAs ENST00000503273, ENST00000462720, and ENST00000480633 are expressed at abnormal levels in hypertriglyceridemic patients with different blood glucose levels and are potential biomarkers of T2DM (114). EPB41L4A-AS1 induced by persistent high glucose inhibits glucose uptake through crotonylation and acetylation of GCN5-mediated proteins, thereby exacerbating the progression of T2DM (115). And, in agreement with the ceRNA hypothesis, lncRNAs have often been found to function as molecular sponges in the study of DM mechanisms. By way of example, in T1DM, four lncRNAs (LINC01278, TRG-AS1, MIAT, and GAS5-AS1) were found to potentially compete with miR-181 to regulate its target genes (116). Moreover, lncRNA KCNQ1OT1 can promote hepatitis C virus-induced β-cell focalization by mediating the miR-223-3p/NLRP3 axis in virally-induced impaired β-cell function (117). LncRNA PTGS2 can impair pancreatic β-cell function by regulating miR-146a-5p and upregulating RBP4, an effect that is reversible (118). And reversal of abnormal lncRNAs can also reverse skeletal muscle IR, such as in the case of lncRNA NONMMUT044897.2 (119). These evidences demonstrated that lncRNAs are located upstream of miRNAs in the regulation of many types of DM and that their use as biomarkers for clinical diagnosis of DM or as potential therapeutic targets is valid and feasible (**Table 2**).

**Table 2:** The role of lncRNAs in diabetes mellitus.

lncRNA	Type of DM	Expression	Effect	Reference
MALAT1	T1DM	Up	Inhibit insulin secretion	(31)
Eif4g2	T2DM	Down	Inhibit oxidative stress	(45)
OIP5-AS1	GDM	Down	–	(111)
HOTAIR	GDM	Up	–	(112)
RPL13p5	GDM	Up	Promote insulin resistance	(113)
ENST00000503273	T2DM	Down	–	(114)
ENST00000462720	T2DM	Up	–	(114)
ENST00000480633	T2DM	Up	–	(114)
EPB41L4A-AS1	T2DM	Up	Inhibit glucose uptake	(115)
LINC01278	T1DM	Down	–	(116)
TRG-AS1	T1DM	Down	–	(116)
MIAT	T1DM	Down	–	(116)
GAS5-AS1	T1DM	Down	–	(116)
KCNQ1OT1	T2DM	Up	Promote the pyroptosis of β-cells	(117)
PTGS2	T2DM	Up	Damage the function of β-cells	(118)
NONMMUT044897.2	T2DM	Up	Promote insulin resistance	(119)

### CircRNAs

**Figure 2:**
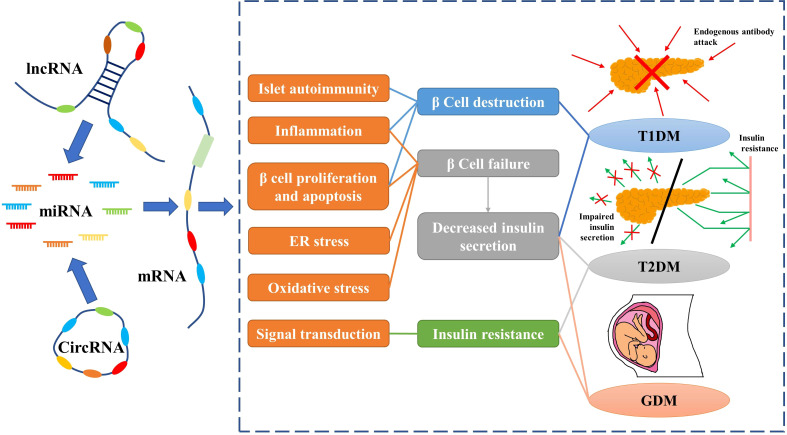
Non-coding RNAs play an important role in diabetes by regulating mRNAs. Red arrows indicate endogenous antibodies and green arrows indicate insulin.

circRNAs are closed-loop RNAs that do not possess polyadenylated tails (120). One benefit of the circular structure of circRNAs is that they are more stable than linear RNAs (121). Although a small number of circRNAs can encode proteins, they are usually classified as ncRNAs; in this review we only discuss the function of circRNAs as ncRNAs (122). According to the ceRNA hypothesis, circRNA possesses abundant MREs and can function as a molecular sponge for miRNAs. Furthermore, some circRNAs have been found to regulate protein-mRNA binding through their function as molecular sponges (123). Considering the stability of circRNAs, they may be more effective than linear ncRNAs as biomarkers for some diseases. As research into the molecular mechanisms of DM has proceeded, circRNAs, along with miRNAs and lncRNAs, have been found to play an important role in diabetes (**Figure 2**).

CircRNAs are expressed at abnormal levels in diabetic patients. CircHIPK3 and circ_0039480 are highly expressed in GDM and T2DM, respectively, and can be used as biomarkers for early diagnosis (124, 125). Circ_0111707 was shown to increase the risk of stress-related T2DM by acting as a molecular sponge for miR-144-3p (126). Circ-Tulp4, which is downregulated in diabetic mouse islets, improves β-cell function by sponging miR-7222-3p and regulating the expression of cholesterol esterification-related genes, sterol O-acyltransferase 1 (SOAT1), and cyclin D1 signaling pathways to promote β-cell adaptation to lipotoxicity (127). CircPIP5K1A as the sponge of miRNA-552-3p regulates glycolipotoxicity-induced inflammation and oxidative damage in rat β-cells *via* Janus kinase 1 (128). Similarly, inhibition of circANKRD36 releases its bound miR-145 to reduce IR and inflammation by inhibiting XBP1 (129). In children with T1DM, circPPM1F expression is elevated and activates macrophages and exacerbates pancreatic injury *via* the circPPM1F -HuR-PPM1F-NF-ΚB axis (130). Inhibition of circLRP6 and circ_0054633, which are upregulated in diabetes, prevents β-cell apoptosis and restores insulin secretion; they also act as molecular sponges for miR-409-3p and miR-9-5p, respectively, in this process (131). Inversely, circ_0060450, which has been found to be highly expressed in children with T1DM, competitively adsorbs miR-199a-5p to upregulate its target gene SHP2, which in turn inhibits macrophage mediated inflammation generated by IFN-I activation of the JAK-STAT1/3 signaling pathway (132). Although circ_0060450 may not prevent the development of pancreatitis and T1DM under the antagonistic effect of other molecules, it sufficiently demonstrates that circRNAs are involved in the self-regulation of the patient’s organism while resisting T1DM. Due to the unmatched stability of circRNAs and the emerging understanding of their roles in DM, they are promising as diagnostic markers or therapeutic targets in the future (**Table 3**).

**Table 3:** The role of circRNAs in diabetes mellitus.

circRNA	Type of DM	Expression	Effect	Reference
circHIPK3	T2DM	Up	–	(124)
circ_0039480	GDM	Up	–	(125)
circ_0111707	T2DM	Up	Increase stress-related T2DM risk	(126)
circ-Tulp4	T2DM	Down	Promote β-cellsadaptation to lipotoxicity	(127)
circPIP5K1A	T2DM	Down	Regulate inflammation and oxidative damage to β-cells	(128)
circANKRD36	T2DM	Up	Promote insulin resistance and inflammation	(129)
circPPM1F	T1DM	Up	Activate macrophages and aggravate islet injury	(130)
circLRP6	T2DM	Up	Promote β-cell apoptosis and insulin secretion injury	(131)
circ_0054633	T2DM	Up	Promote β-cell apoptosis and insulin secretion injury	(131)
circ_0060450	T1DM	Up	Inhibit macrophage mediated inflammation	(132)

### Predicting the role of ncRNAs in DM by bioinformatics

**Figure 3:**
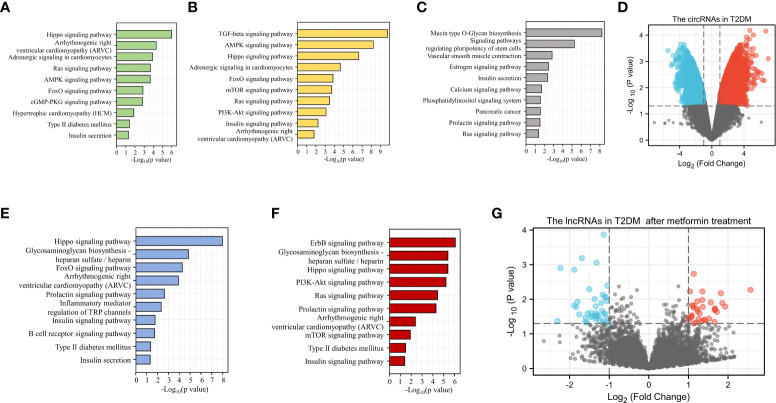
Bioinformatic analysis of the potential role of ncRNAs in DM. KEGG analysis of the target genes of miRNAs up-regulated **(A)** or down-regulated **(B)** in T1DM. KEGG analysis of the target genes of differentially expressed miRNAs in T2DM **(C)**. Differential expression of circRNA in T2DM **(D)**. KEGG analysis of the target genes for up-regulated **(E)** or down-regulated **(F)** miRNAs in GDM. LncRNAs with altered expression in T2DM patients after metformin treatment **(G)**.

Because sequencing has become a key tool in studies related to the mechanisms of diabetes, we were able to use sequencing results from GEO to analyze ncRNAs in DM (https://www.ncbi.nlm.nih.gov/geo/). With this sequencing data, we performed KEGG analysis of target genes of miRNAs differentially expressed in patients with various types of DM and predicted the potential role of these miRNAs in the development of DM and CVD (**Figures 3A–C, E, F**) . We observed a large number of circRNA abnormalities in T2DM patients (**Figure 3D**). Additionally, we observed changes in the expression of lncRNAs in T2DM patients treated with metformin, suggesting that lncRNAs are involved in the therapeutic effect of metformin on DM (**Figure 3G**). Currently, the role of a proportion of ncRNAs have emerged gradually. The aberrant expression of ncRNAs in DM patients and the modulatory effects of anti-DM drugs on ncRNAs are suggesting that ncRNAs may provide greater promise for the diagnosis and treatment of diabetes and cardiovascular disease.

## NcRNAs in diabetic cardiovascular disease

### Diabetes mellitus and cardiovascular disease

CVD is the most common and dangerous complication of DM, with more than 50% of T2MD patients die from it (133). DM is considered to be one of the main causative factors of CVD. Obesity is also a key risk factor in both DM and CVD, a commonality that makes the link between diabetes and cardiovascular disease even stronger (134). Previous evidence suggests that DM increases the chance of CVD by accelerating atherosclerotic lesions (135). In contrast to other tissues, the vascular endothelium is more sensitive to blood glucose, which makes it a major target of hyperglycemic injury (136). Metabolic disorders caused by IR or abnormal insulin levels can cause vascular endothelial dysfunction (137) and the prolonged persistence of hyperglycemic state can lead to the accumulation of intracellular reactive oxygen species. In response to oxidative stress, the patients’ IR increases and contributes to increased vascular permeability (138). Vascular destruction in diabetes patients may be caused by increased fluxes of polyol pathways, diacylglycerol, aldose reductase, late glycosylation end products, protein kinase C (PKC), and fructose 6-phosphate (139). The late glycosylation end products of hyperglycemia severely disrupt the protective effect of nitric oxide (NO) on the endothelium, and its effects on macrophages, monocytes, and vascular smooth muscle cells are widespread. This makes the inflammatory response and oxidative stress in the endothelial system more severe (140). Considering the correlation between CVD and DM and the corresponding health risk of concomitance, prevention and treatment of CVD is the most critical aspect in the management of patients with DM.

### NcRNAs and diabetic CVD

**Figure 4:**
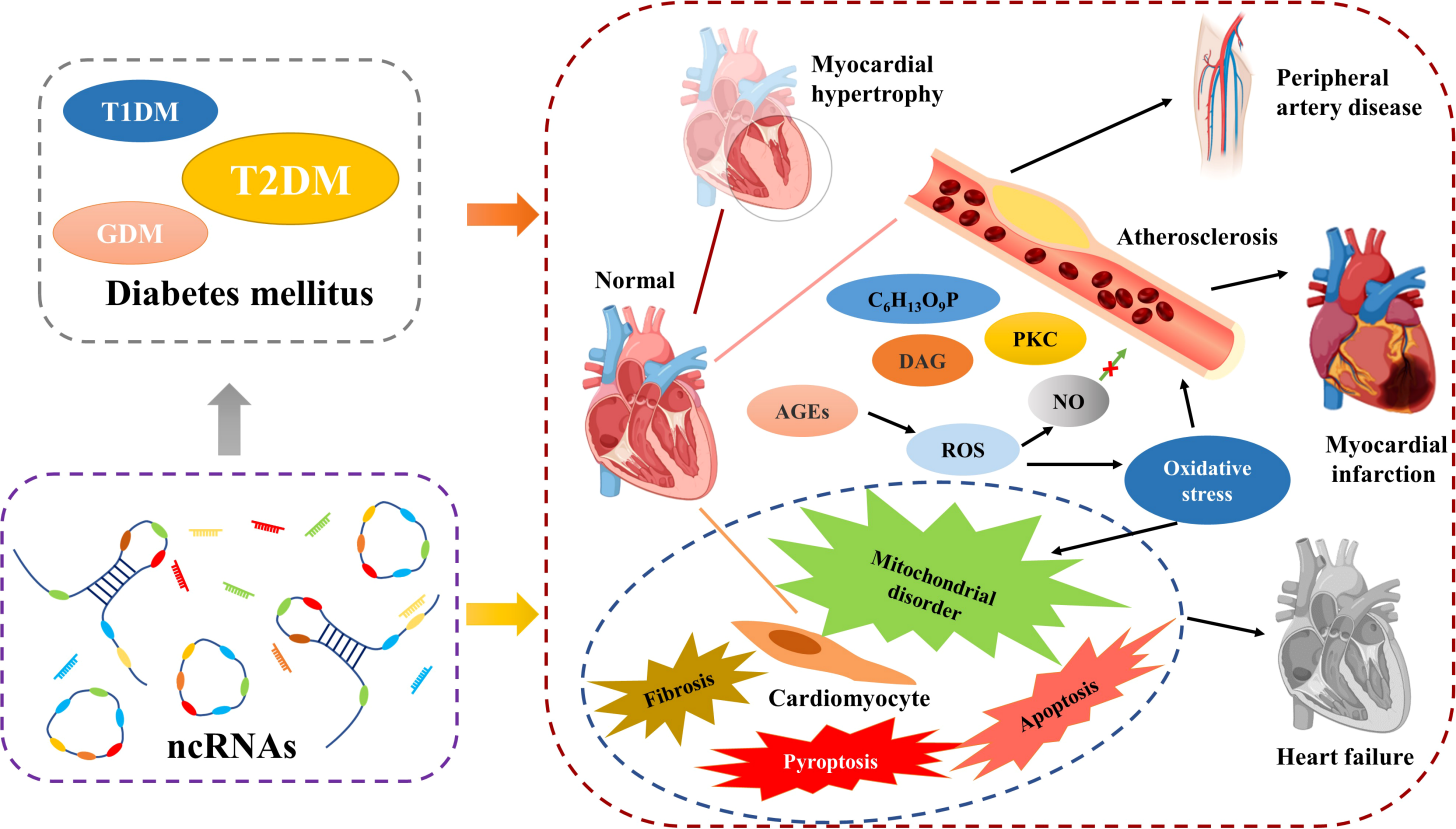
NcRNAs are participating in the development of diabetic CVD.

As in DM, ncRNAs also play an important role in the development of diabetic CVD. The main phenomena of uropathy cardiovascular disease are cardiac hypertrophy, atherosclerosis, heart failure. In addition, cardiomyocyte-related apoptosis, scorch death, autophagy, fibrosis, mitochondrial dysfunction and oxidative stress, inflammatory response are also important. As the understanding of the mechanisms of diabetic CVD has become more advanced, researchers have found that ncRNAs are extensively involved in these processes (**Figure 4**).

A study showed that miR-30c expression was reduced in DM patients, that its absence induced cardiac hypertrophy cardiac hypertrophy (141). Recent studies have shown that in GDM, miR-195-5p inhibits vascular endothelial growth factor A to promote endothelial dysfunction (142). And in another study, silencing miR-195 attenuated diabetic cardiomyopathy in mice (143). In T2DM, downregulation of erythroid miR-210 induces endothelial dysfunction (144). Previous work has found that knocking down circ_0071269 prevents cardiomyopathy injury through the miR-145/gasdermin A axis (145), whereas miR-424-5p affects cardiovascular health through anti-angiogenesis in T1DM (146). The lncRNA ZFAS1 can act as a molecular sponge for miR-150-5p, and, when ZFAS1 is inhibited it, can reduce iron death and combat diabetic cardiomyopathy by activating CCND2 (147). CircCDR1as promotes cardiomyocyte apoptosis in diabetic cardiomyopathy by activating the hippo signaling pathway; in contrast, circHIPK3 is downregulated in diabetic cardiomyopathy and protects cardiomyocytes from high-glucose-induced apoptosis when overexpressed (148, 149). Reducing the expression of lncRNA TINCR and MIAT inhibit cell scorching and alleviate diabetic cardiomyopathy, the former by enhancing the mRNA stability of NLRP3 and the latter by acting as a molecular sponge for miR-214-3p (150, 151). miR-135b and miR-30a-5p are expressed at lower level in DM, and when their expression levels are increased, they can be used to treat diabetic cardiac fibrosis by inhibiting cardiomyocyte scorching (152, 153). miR-340-5p targets Mcl-1 to mediate diabetes-induced oxidative stress in cardiomyocytes (154). Inhibition of miR-378a restores cardiac function by rescuing ATP synthase in the diabetic heart (155). As cardioprotective effects were found in T1DM patients treated with the classical DM drug metformin, such effects may be caused by downregulated miR-222, miR-195, and miR-21a (156). In conclusion, ncRNAs play an important role in diabetic CVD and therefore are good biomarkers for diabetic CVD and may also be key targets for the treatment of diabetic CVD (**Table 4**).

**Table 4:** The role of ncRNAs in diabetic CVD.

ncRNA	Type of DM	Expression	Effect	Reference
miR-30c	-	Down	Low expression can induce myocardial hypertrophy	(141)
miR-195-5p	GDM	Up	Promote endothelial dysfunction	(142)
miR-195	-	Up	Low expression reduces diabetic cardiomyopathy	(143)
miR-210	T2DM	Down	Low expression promotes endothelial dysfunction	(144)
circ_0071269	-	Up	Low expression can prevent cardiomyopathy injury	(145)
miR-424-5p	T1DM	Up	Anti-angiogenesis	(146)
lncRNA ZFAS1	-	Up	Low expression can alleviate diabetes cardiomyopathy	(147)
CircCDR1as	-	Up	Promote the apoptosis of cardiomyocyte	(148)
CircHIPK3	-	Down	Protect cardiomyocytes from apoptosis	(149)
lncRNA TINCR	-	Up	Promote the pyroptosis of cardiomyocytes	(150)
lncRNA MIAT	-	Up	Promote the pyroptosis of cardiomyocytes	(151)
miR-135b	-	Down	Protect cardiomyocytes from pyroptosis	(152)
miR-30a-5p	-	Down	Protect cardiomyocytes from pyroptosis	(152)
miR-340-5p	-	Up	Cause oxidative stress in cardiomyocytes	(154)
miR-378a	-	Up	Reduce ATP synthase activity in the heart	(155)
miR-222	T1DM	Up	It is related to the protective effect of metformin on the heart	(156)
miR-195	T1DM	Up	It is related to the protective effect of metformin on the heart	(156)
miR-21a	T1DM	Up	It is related to the protective effect of metformin on the heart	(156)

## Discussion

As a chronic disease with no cure, a high incidence, and many associated health complications, DM presents a high health risk. DM not only brings inconvenience to the lives of patients, causing bodily or even life-threatening damage, but also imposes a huge burden on patients’ families and the social health care system. For these reasons, work on the diagnosis and treatment of DM has been an important area of interest in medical research, and advances in this field will directly or indirectly reduce the risk of DM complications like diabetic CVD.

One of the traditional methods of treatment for T1DM is the administration of exogenous insulin supplementation (157, 158). However, islet transplantation is one of the conventional T1DM treatments, and appears to be longer lasting. Clinical outcome assessment is important in this treatment approach, and miR-375, an ncRNA, whose levels represent post-transplant islet activity, can be used as a reliable biomarker to assess prognosis (159, 160). Furthermore, several new therapies have been developed in recent years, in which ncRNAs also play an important role. For stem cell-based therapies that regenerate β-cells through bone marrow transplantation of stem cells, miR-212/132 can play an adjuvant role by promoting the induction of stem cell differentiation to β-cells (105, 161, 162). While immune tolerance therapy can slow the progression of T1DM by suppressing the patient’s immune system, it is costly and has the potential to induce adverse effects (163). A recent study reported that islet non-β-cells can secrete insulin after reprogramming, which brings a new idea to the treatment of T1DM, however, it is still not widely used in the clinic (164).

Metformin, a first-line drug for T2DM treatment, has been found to reduce elevated miR-221/222/223 in DM in a dose-dependent manner, suggesting that regulation of ncRNA expression may be one of the key mechanisms of metformin in DM treatment (165, 166). There are several new anti-T2DM drugs in development that have a better safety profile and can prevent complications (167). Although the development of these drugs tends to be aimed at personalization for the patient, there are still significant limitations to reaching a true personalization that most patients can obtain.

As a key factor driving the development of complications such as DM and CVD, ncRNA plays an important role in several aspects as β-cell failure, insulin secretion, and IR. The function of β-cells is regulated by ncRNAs and sometimes β-cells function through ncRNAs. High expression of miR-212-5p inhibits insulin secretion in β-cells (168). While β-cells enhance insulin sensitivity and control glucose homeostasis through the miR-26a and miR-29 family (101, 169).Various evidence now shows that ncRNAs such as miR-1249-3p, lncRNA PTGS2, and circ-Tulp4 can alleviate DM by reversing the links they are participating in through restoration of their expression levels; ncRNAs thus have full potential to act as therapeutic targets. With the help of gene editing technology, it may also be possible to use gene therapy to restore the expression of abnormal ncRNAs in patients in the long term (170). A recent study developed a tool for RNA delivery that targets β-cells (171). In addition, various carriers, including liposomes and exosomes, have been found to be useful for delivering RNA (172, 173). The delivery system of ncRNAs might have potential application in clinical treatment of DM and CVD. Furthermore, benefiting from the diversity of gene editing tools, it may be possible in the future to truly personalize the treatment of diabetic patients.

Insulin therapy in GDM is a treatment recognized as harmless but its dosage must be strictly controlled. A study showed that miR-27 inhibits Akt phosphorylation by targeting Pdpk1 and Pik3r1 in the context of IR and that insulin treatment promotes miR-27 expression, thus deepening IR (174). However, while it was demonstrated in this research that treatment with metformin inhibited miR-27 expression to reduce its effects, the potential side effects of metformin are unclear and safety remains uncertain. This result suggests that regulation of ncRNA expression may be one of the key roles of traditional diabetes therapeutics. Moreover, the regulation of ncRNAs expression pattern may enhance the traditional treatments for DM.

Common diagnostic markers for DM include fasting plasma glucose (FPG), oral glucose tolerance (OGT), and glycated hemoglobin (HbA1c), etc. (175). However, these indicators are not particularly effective in the early diagnosis of DM. Several molecules have been used as novel molecular biomarkers for DM. The main biomarkers of T1DM include C-peptides that can represent insulin production, and autoantibodies against endogenous islet antigens such as GAD, ZnT8, insulin (23, 176). In T2DM, inflammatory systemic markers such as Glycosylated acetyls (GlycA), Advanced Glycation End Products (AGEs) are effective biomarkers (177, 178). In addition, based on the presence of higher levels of oxidative damage in DM, lipid peroxidation markers such as isoprostane, protein oxidation markers such as advanced oxidation protein products (AOPPs), and oxidative DNA damage biomarkers such as 8-OHdG can also be utilized for the diagnosis of DM (179). Due to the fact that GDM can have various long-lasting effects on both the patient and the fetus, prevention of GDM is crucial. The guidelines laid about by the American College of Obstetricians and Gynecologists (ACOG) recommend screening in high-risk groups. Early screening is usually performed at 24-28 weeks of gestation and includes a fasting glucose and oral glucose tolerance test (OGTT) (180). However, abnormalities of ncRNAs often appear early in the course of symptoms. One study successfully diagnosed GDM by miRNA early in pregnancy (around 10 weeks) and required a much shorter fasting time before sampling (at least 4 hours compared to 10-16 hours for OGTT (181). Thus, screening with ncRNAs as biomarkers has undeniable advantages: Firstly, ncRNA changes can be more sensitive to predict GDM at an earlier stage of pregnancy and thus allow better intervention. Secondly, the ncRNA assay is more convenient and stable, enabling shorter fasting time and reduction in maternal discomfort.

CVD, as the most dangerous complication of DM, is the leading cause of death in patients with T1DM and T2DM (182). DM can cause numerous negative effects on the patient’s internal environment, such as hyperlipidemia, hyperglycemia, higher oxidative stress, and persistent inflammation (183, 184). These factors have a critical role to play in the development of diabetic CVD (185–189). Diabetic CVD can be broadly divided into cardiomyopathy and vascular disease (190). Diabetic cardiomyopathy mainly includes increased cardiomyocyte death triggered by the DM environment and cardiomyocyte dysfunction caused by abnormal myocardial mitochondrial calcium ion handling (191). Atherosclerosis is the most common form of diabetic vascular disease. The high levels of AGE caused by DM and the stimulation of oxidative stress cause endothelial dysfunction and trigger a sustained inflammatory response that eventually leads to atherosclerosis (192). NcRNAs play an important role in the process of DM-induced CVD. As previously described, aberrantly expressed ncRNAs promote cardiomyocyte death or endothelial injury (142, 148, 149). NcRNAs bring new perspectives for the prevention and treatment of diabetic CVD. From the perspective of prevention, ncRNAs can be used to screen or treat DM to avoid the risk of CVD. In addition, recent studies support the use of ncRNAs as therapeutic targets for CVD, for example, inhibition of ZFAS1, TINCR, and MIAT can reduce abnormal programmed cardiomyocyte death (147, 150, 151).

## Conclusion

DM poses a major concern to human health. Although it is not fatal as a chronic disease, many complications it causes can have serious consequences and even lead to death. CVD, in particular, is the most lethal of the many complications of DM. Evidences suggested that ncRNAs are involved in the development of DM and can be used as biomarkers for the diagnosis of DM. This indicates that ncRNAs have potential application in the future for clinical treatment of DM and associated CVD.

## Author contributions

CSL, DW, and DL wrote the manuscript. CSL, ZJ, YG, LS, RL, MC, and CL collected the references and prepared figures. All authors contributed to the article and approved the submitted version.

## Funding

This work was supported by the Jilin Health Commission Program under Grant 2020J05S, the Fundamental Research Funds for the Central Universities under Grant 2019JCKT-70, the Jilin Education Department Program under Grant JJKH20200950KJ, and the Jilin Scientific and Technological Development Program under Grant 20220505033ZP, 202002006JC and 20210101010JC. The scientific research project of key laboratories of colleges and universities in Jilin Province [2019] No. 004.

## Conflict of interest

The authors declare that the research was conducted in the absence of any commercial or financial relationships that could be construed as a potential conflict of interest.

## Publisher’s note

All claims expressed in this article are solely those of the authors and do not necessarily represent those of their affiliated organizations, or those of the publisher, the editors and the reviewers. Any product that may be evaluated in this article, or claim that may be made by its manufacturer, is not guaranteed or endorsed by the publisher.
